# Self-assessment of risk of dysphagia and clinical-functional vulnerability in older adults with a history of leprosy

**DOI:** 10.1590/2317-1782/e20240192en

**Published:** 2025-12-01

**Authors:** Maria Clara Rocha, Aline Mansueto Mourão, Jéssica Danielle Santos de Jesus, Laélia Cristina Caseiro Vicente, Adriane Mesquita de Medeiros

**Affiliations:** 1 Programa de Pós-graduação em Ciências Fonoaudiológicas, Departamento de Fonoaudiologia, Universidade Federal de Minas Gerais – UFMG - Belo Horizonte (MG), Brasil.

**Keywords:** Leprosy, Elderly, Frailty, Swallowing, Swallowing Disorders, Dental Prosthesis, Institutionalization

## Abstract

**Purpose:**

To relate self-assessed dysphagia risk to clinical-functional vulnerability, age, sex, dentition, education, and institutionalization of older adults with a history of leprosy, and to assess the swallowing quality of life of those at risk.

**Methods:**

Cross-sectional study with 117 older people. The inclusion criteria were being 60 years or older, with a history of leprosy, and no history of mental disorders, cognitive impairment due to stroke, or dementia syndrome. The study obtained information such as sex, age, education, institutionalization, and dentition, and used the following protocols: Clinical-Functional Vulnerability Index (IVCF-20), Eating Assessment Tool (EAT-10), and Quality of Life in Swallowing Disorders (SWAL-QOL). The association between self-assessed dysphagia risk in older adults, a history of leprosy, and the other variables was verified with Pearson's chi-square and Fisher's exact tests, with a 5% significance level.

**Results:**

There was a predominance of non-institutionalized older women aged 80 to 99 years, with incomplete elementary education, using dentures. According to self-assessment, 22 older adults (18.8%) were at risk of dysphagia, and the most compromised quality of life domain was “eating duration.” The risk of dysphagia was statistically associated with age and clinical-functional vulnerability.

**Conclusion:**

Being over 80 years old and being considered frail increases the chance of older people with a history of leprosy having a self-assessed risk of dysphagia. The greatest impairment in the quality of life was related to “eating duration”.

## INTRODUCTION

Leprosy is a chronic, infectious, dermatological, neurologic disease, subject to mandatory notification and investigation, which affects the superficial nerves of the skin and peripheral nerve trunks^(1)^, including the cranial nerves^(2)^. The etiological agent is Mycobacterium leprae, primarily transmitted through the upper airways, causing sensory, motor, and autonomic changes^(1)^. It has a highly disabling effect and affects individuals of both sexes and all ages. Treatment is free, offered by the Brazilian Unified Health System (SUS), and administered through a combination of drugs called polychemotherapy (PCT)^(1)^.

Early diagnosis and appropriate treatment are the main ways to prevent physical disabilities and impairments caused by the disease. However, once these have already developed, care should be directed toward maintaining or improving the patients’ physical, socioeconomic, and emotional conditions^(1,3)^. The Global Leprosy Strategy (2016-2020), created by the World Health Organization (WHO), aims to reduce the global and local burden of leprosy and eliminate existing stigma, discrimination, and social exclusion^(3)^.

Although the number of people infected with leprosy has decreased over the last decade^(3)^, it remains a major health problem^(4)^. In 2020, 127,396 cases were recorded worldwide, with Brazil ranking second only to India^(4)^. Preliminary data for 2022 show that 14,962 new cases of leprosy were diagnosed in Brazil. Between 2017 and 2021, 119,698 new cases of leprosy were diagnosed in Brazil. Of this total, 66,613 new cases occurred in males, corresponding to 55.7% of the total. This predominance was observed in most age groups and years of evaluation, with a higher frequency in individuals aged 50 to 59 years and with incomplete elementary education (40.9%)^(4-6)^. The prevalence of the disease is directly linked to aging^(6)^, physical disabilities^(7)^, and sociodemographic, economic, health, and environmental issues^(3)^. Therefore, it is important to identify vulnerable groups and to implement intersectoral management that ensures equity in access to public policies for social inclusion, health services, health education, and income, strengthening leprosy control actions^(1,3)^.

Leprosy can affect the structures of the stomatognathic system and vocal tract, compromising communication and swallowing functions^(8-12)^. Studies indicate the involvement of the cranial nerves responsible for the swallowing process, such as the trigeminal, facial, vagus, spinal accessory, and hypoglossal nerves, with the trigeminal and facial nerves being the most affected in people with leprosy^(2,8)^. Furthermore, the disease can affect the laryngeal mucosa and develop symptoms such as dry cough, globus sensation in the throat, odynophagia, stridor, dysphagia, dysphonia, aphonia, and dyspnea^(11-13)^. Although symptoms appear in more advanced stages of the disease^(11,13)^, most individuals have efficient swallowing^(12)^. It is reported that iatrogenic factors, advanced age, and early intervention in treatment can influence the presence and degree of dysphagia^(12,13)^.

Aging and dysphagia are potential risk factors for the development and progression of frailty^(10,14-16)^. Thus, professionals can contribute to these people’s quality of life by using targeted protocols^(9)^ to identify dysphagia signs and symptoms in older adults with a history of leprosy, promoting preventive and rehabilitative actions to reduce complications such as pulmonary aspiration, aspiration pneumonia, malnutrition, and dehydration, which lead to functional and health impairment^(8-10,12)^.

The study hypothesizes that self-assessed dysphagia risk in older adults is related to a history of leprosy and clinical-functional vulnerability, age, sex, dentition, education, and institutionalization. Therefore, it is necessary to define the older people who are at risk of dysphagia and how swallowing impacts their quality of life to expand care for older adults, given the functional losses resulting from leprosy and aging.

Thus, this study aimed to analyze the relationship between self-assessment of dysphagia risk and clinical-functional vulnerability, age, sex, dentition, education, and institutionalization of older people with a history of leprosy, and to verify the quality of life in swallowing for those at risk.

## METHODS

This cross-sectional, analytical, observational study was approved by the research ethics committees of the Minas Gerais Hospital Foundation (FHEMIG) and the Federal University of Minas Gerais (UFMG) under approval number 2,373,001. All participants gave their consent through an informed consent form.

The study comprised 117 older adults; the inclusion criteria were being 60 years or older, with a history of leprosy, and no history of mental disorders, cognitive impairment due to stroke, or dementia syndrome. The exclusion criteria were older individuals with suspected cognitive impairment according to the Mini-Mental State Examination (MMSE), according to their level of education^(17)^.

Data were collected at an institution in the metropolitan region of Minas Gerais. The institution opened in 1931, when compulsory hospitalization and isolation of patients were the measures adopted for leprosy treatment and prophylaxis, a practice that ceased in the late 1980s. Most older adults in this study currently live in their own homes, while the most fragile ones and those who have lost family and social ties live in the institution's long-term care facilities for older people. Specialized care is offered at outpatient clinics, homes, and long-term care facilities, with the mission of primarily caring for older adults with a history of leprosy resulting from the period of compulsory hospitalization. The team consists of a speech-language-hearing pathologist, physical therapist, occupational therapist, nursing staff, social workers, psychologists, and nutritionists.

The primary data collection for the study occurred in two stages, both during the same collection period. The first extracted current data from the institution's medical records, including personal information such as age, sex, edentulism, denture fitting, and institutionalization. The second stage applied selected protocols. All study participants were evaluated by a speech-language-hearing pathologist from the institution's rehabilitation team.

The study used the Clinical-Functional Vulnerability Index (IVCF-20), a questionnaire validated in Brazil that considers multidimensional aspects of the health status of older adults. The protocol has 20 questions distributed across eight sections: age, self-perceived health, functional disabilities, cognition, mood, mobility, communication, and multiple comorbidities. Each section has a specific score, totaling a maximum of 40 points. The higher the score, the greater the older adult's clinical-functional vulnerability. The IVCF-20 classification of older adults is presented in three categories: robust older adults (with a total score between 0 and 6), older adults at risk of frailty (with a score between 7 and 14), and frail older adults (with a score equal to or greater than 15)^(18)^. The IVCF-20 is part of the institution's routine rehabilitation service, administered by a team comprising speech-language-hearing pathologists, physical therapists, occupational therapists, psychologists, social workers, nutritionists, and nurses. The evaluators underwent prior training in 2016 with the team that participated in the development of the IVCF-20. The data from this protocol that were included in the study referred to assessments conducted during the same period as the primary data collection for this research.

Next, the protocol for self-assessment of dysphagia risk and swallowing-related quality of life was applied. The Portuguese version of the Eating Assessment Tool (EAT-10)^(19,20)^ aims to self-assess dysphagia risk with 10 simple questions: three from the functional domain, three from the emotional domain, and four from the physical domain. The maximum total score is 40 points. Scores equal to or greater than 3 points indicate the risk of dysphagia. The calculation is simple and performed by adding the results of each item.

The Quality of Life in Swallowing Disorders (SWAL-QOL) self-assessment instrument^(21)^ was applied in its validated Portuguese version^(22)^ to older adults at risk for dysphagia according to the EAT-10 to identify the impact of self-assessed dysphagia risk on quality of life. This questionnaire has 44 questions that assess 11 domains: swallowing as a burden, eating desire, eating duration, symptom frequency, food selection, communication, fear of eating, mental health, social health, sleep, and fatigue. Responses related to frequency and degree of veracity are marked using a Likert scale ranging from 1 to 5, assigning the following points: 1 = 0, 2 = 25, 3 = 50, 4 = 75, and 5 = 100. The score ranges from 0 to 100, with lower scores indicating worse swallowing-related quality of life. The scores for each response within each domain are added together, and the result is divided by the number of questions in the domain being analyzed. The final score is the sum of the scores obtained in each domain divided by the total number of domains.

Data analysis was descriptive, using absolute and relative frequency distributions. Pearson's chi-square test and Fisher's exact test were used to analyze the association between self-assessed dysphagia risk and explanatory variables. Variables with statistical significance in the association tests (age and clinical-functional vulnerability) were included in the univariate logistic regression analysis to estimate the odds ratio. A 5% significance level was considered for all analyses. Data were processed and analyzed using STATA version 13.

## RESULTS

The study included 117 older men and women, with a predominance of females (54.7%), aged 80 to 99 years (52.99%), with incomplete elementary education (75.2%). Also, 18 (15.4%) were institutionalized, 90 (77.0%) did not have natural teeth, and 44 (37.6%) used partial or complete dentures. Regarding functional vulnerability, 37.6% of the older adults were considered robust, 35.0% at risk of frailty, and 27.5% frail (Table 1).

**Table 1:** Description of sociodemographic characteristics, dental conditions, and social vulnerability index of older adults with a history of leprosy (n=117)

Variables	Older people at risk of dysphagia	Older people not at risk of dysphagia	Total	p-value
	n (%)	n (%)		
**Age**				
64 to 79 years	5 (22.7)	50 (52.6)	55 (47.0)	0.011*^a^
80 to 99 years	17 (77.3)	45 (47.4)	62 (53.0)	
**Sex**				
Females	10 (45.5)	54 (56.8)	64 (54.7)	0.33*
Males	12 (54.5)	41 (43.2)	53 (45.3)	
**Institutionalization**				
Yes	5 (22.7)	13 (13.7)	18 (15.4)	0.29*
No	17 (77.3)	82 (86.3)	99 (84.6)	
**Education level**				
No education	7 (31.8)	17 (17.9)	24 (20.5)	0.55**
middle school incomplete	15 (68.2)	73 (76.8)	88 (75.2)	
middle school graduate	0 (0.0)	1 (1.1)	1 (0.9)	
high school incomplete or graduate	0 (0.0)	4 (4.2)	4 (3.4)	
**Natural teeth**				
Yes	7 (31.8)	20 (21.1)	27(23.1)	0.28*
No	15 (68.2)	75 (78.9)	90 (76.9)	
**Well-fitted dentures**				
Yes	5 (22.7)	39 (41.1)	44 (37.6)	0.13*
No No denture use	12 (54.5) 5 (22.7)	31 (32.6) 25 (26.3)	43 (36.8) 30 (25.6)	
**Clinical-Functional Vulnerability Index (IVCF-20)**				
Robust	4 (18.2)	40 (42.1)	44 (37.6)	0.05*^a^
At risk of frailty	8 (36.4)	33 (34.7)	41 (35.0)	
Frail	10 (45.4)	22 (23.2)	32 (27.4)	

* Pearson's chi-square test;

** Fisher's exact test

a p -value ≤ 0.05

It was found that only age and IVCF-20 were associated in older adults at risk of dysphagia (Table 1).

According to the EAT-10, 22 older people (18.8%) were self-assessed at risk of dysphagia. The most reported symptoms were “I need to force myself to swallow medicine”, “I get food stuck in my throat”, and “I cough when I eat” (Table 2).

**Table 2:** Description of EAT-10 responses (n=117)

Variables	Total
n (%)	
**My swallowing problem has caused me to lose weight**	
No problem	115 (98.30%)
Small problem	0 (0.00%)
Medium problem	2 (1.70%)
Great problem	0 (0.00%)
Severe problem	0 (0.00%)
**My swallowing problem interferes with my ability to go out for meals**	
No problem	110 (94.00%)
Small problem	0 (0.00%)
Medium problem	3 (2.60%)
Great problem	2 (1.70%)
Severe problem	2 (1.70%)
**Swallowing liquids takes extra effort**	
No problem	109 (93.15%)
Small problem	1 (0.85%)
Medium problem	5 (4.30%)
Great problem	1 (0.85%)
Severe problem	1 (0.85%)
**Swallowing solids takes extra effort**	
No problem	105 (89.75%)
Small problem	1 (0.85%)
Medium problem	6 (5.15%)
Great problem	4 (3.40%)
Severe problem	1 (0.85%)
**Swallowing pills takes extra effort**	
No problem	95 (81.20%)
Small problem	0 (0.00%)
Medium problem	18 (15.40%)
Great problem	3 (2.55%)
Severe problem	1 (0.85%)
**Swallowing is painful**	
No problem	115 (98.30%)
Small problem	0 (0.00%)
Medium problem	2 (1.70%)
Great problem	0 (0.00%)
Severe problem	0 (0.00%)
**The pleasure of eating is affected by my swallowing**	
No problem	113 (96.60%)
Small problem	0 (0.00%)
Medium problem	3 (2.55%)
Great problem	1 (0.85%)
Severe problem	0 (0.00%)
**When I swallow, food sticks in my throat**	
No problem	99 (84.61%)
Small problem	4 (3.41%)
Medium problem	8 (6.83%)
Great problem	4 (3.41%)
Severe problem	2 (1.70%)
**I cough when I eat**	
No problem	89 (76.05%)
Small problem	2 (1.70%)
Medium problem	14 (12.00%)
Great problem	9 (7.70%)
Severe problem	3 (2.55%)
**Swallowing is stressful**	
No problem	113 (96.60%)
Small problem	0 (0.00%)
Medium problem	4 (3.40%)
Great problem	0 (0.00%)
Severe problem	0 (0.00%)
**Risk of dysphagia - EAT**	
Yes (cutoff: > 2 points)	22 (18.80%)
No (cutoff: up to 2 points)	95 (81.20%)

**Caption:** EAT-10 = Eating Assessment Tool

All participants considered at risk for dysphagia completed the quality-of-life questionnaire (SWAL-QOL). The highest mean scores were in the Communication (88.0%) and Social (86.8%) domains, indicating that swallowing quality of life is not a problem for older adults with self-assessed dysphagia risk. The greatest impairment in the swallowing quality of life was related to eating time (19.3%). The lower mean score showed that older adults take longer to eat than other people and perceive that they take a long time to eat (Table 3).

**Table 3:** Description of the median, mean, and standard deviation of Swal-QOL scores of older people with self-assessment of the risk of dysphagia

Domains	Older people at risk of dysphagia	Mean (standard deviation)
	Median	
Swallowing as a burden	62.5	61.9 (28.2)
Eating desire	66.7	64.4 (29.5)
Eating duration	0	19.3 (32.4)
Symptom frequency	79.5	74.4 (10.9)
Food selection	81.3	79.5 (23.0)
Communication	100.0	88.0 (21.2)
Fear of eating	62.5	65.0 (26.8)
Mental health	80.0	73.8 (21.6)
Social	92.5	86.8 (16.2)
Sleep	56.3	63.0 (26.5)
Fatigue	75.0	73.8 (17.1)
SWAL-QOL total score	71.9	67.9 (10.6)

**Caption:** Swal-QOL = Quality of Life in Swallowing Disorders

The univariate logistic regression analysis confirmed the statistical association between self-assessed risk of dysphagia and age and clinical-functional vulnerability (Table 4).

**Table 4:** Univariate logistic regression model of the association of self-assessed risk of dysphagia with age and IVCF-20

	OR (95% CI)
**Age**	
64 to 79 years	1
80 to 99 years	3.78 (1.29-11.07)*
	
**IVCF-20**	
Robust	1
At risk of frailty	2.42 (0.67-8.77)
Frail	4.55 (1.28-16.20)*

* p-value ≤ 0.05

**Caption:** IVCF-20 = Clinical-Functional Vulnerability Index; OR = Odds Ratio; 95% CI = 95% confidence interval

The result shows that frail older people are 4.55 times more likely to self-assess their risk of dysphagia than those with a robust clinical-functional index. Those with a more advanced age are 3.78 times more likely to self-assess their risk of dysphagia (Table 4). The multivariate model was not performed with the two statistically significant variables, as the analysis between age and IVCF-20 showed that their functional vulnerability was related to age. Those aged 80 to 99 years are more likely to be frail (OR = 4.94/95% CI = 1.83-13.31) or at risk of frailty (OR = 2.73/95% CI = 1.13-6.58) than those aged 64 to 79 years.

## DISCUSSION

This study showed that most older people with a history of leprosy did not self-assess their risk of dysphagia. However, self-assessment of dysphagia risk was associated with age and a higher clinical-functional vulnerability index measured by the IVCF-20. The most compromised swallowing quality of life domain was “eating duration.”

Current epidemiological data in Brazil indicate a higher prevalence of leprosy in adults^(4)^, in the age group close to older adults^(6)^, which contributes to their increased susceptibility to oral and laryngopharyngeal changes^(12,13)^ and clinical-functional vulnerability^(10)^. Although older people have been diagnosed with and treated for leprosy in the past, before old age, they live with the chronic sequelae resulting from the disease^(1,3)^ and consequent loss of functioning due to aging^(7,15)^.

There was a predominance of females, aged 80 to 99 years, with a low level of education, corroborating the literature^(7,23,24)^. Self-assessed risk of dysphagia was not statistically associated with sex and education in the present study, as observed in other studies, which did not find this relationship with sex in healthy older adults^(25)^ or with education in institutionalized older women^(26)^. In contrast, the literature observes dysphagia in women with low education^(15,24)^. This finding can be explained by the greater demand of females for health services and the cultural, behavioral, educational, and lifestyle aspects involved^(27)^.

The EAT-10 showed that 18.8% of older people with a history of leprosy self-assessed at risk of dysphagia, a value below that of another study with a similar population, but with clinical evaluation of swallowing^(12)^.

Findings related to dysphagia in older adults without a diagnosis of leprosy showed a risk of dysphagia in independent (25.1%), dependent (53.8%)^(28)^, healthy (38.5%)^(25)^, hospitalized (31.1%)^(29)^, and frail (58%)^(15)^ individuals. This explains why, despite the high number of patients with a history of leprosy and efficient swallowing, many older individuals, regardless of their clinical conditions, have mild to moderate dysphagia^(12)^, which may be associated with aging and pathological processes^(15,30)^. Different protocols and classifications for assessing dysphagia may justify the differences found between studies. However, early diagnosis and treatment of the disease reduce complications and loss of functioning, which may explain the result.

Most participants lacked natural teeth, and few used dentures. Although the study found no statistical association between self-assessed dysphagia risk and dental conditions, studies indicate a relationship between dysphagia and the number of teeth^(15,26)^. Korean older adults had a 61.6% risk of dysphagia, with reduced chewing ability being one of the associated factors^(24)^. A study of institutionalized older people found increased chewing time^(23)^. Therefore, the loss of natural teeth, the use of poorly fitted dentures^(15,23,24,26)^, and injuries to the cranial nerves responsible for this function^(2,8)^ can interfere with the oral preparation of the bolus and the swallowing process, impacting digestion, communication, social interaction, and quality of life.

All participants in this study underwent compulsory isolation for leprosy treatment. After mandatory isolation ended, many returned to their homes and resumed their usual daily activities. There was no statistical significance in the self-assessed risk of dysphagia in institutionalized older adults. However, a study of 23 institutionalized older Brazilian individuals found swallowing disorders associated with aging in 82.6% of participants^(23)^. Institutionalized older individuals in another study had a 63.3% risk of dysphagia, related to factors such as medication use, number of teeth, and feeding dynamics^(26)^. Even though this study lacked results regarding nutritional status, the literature indicates an increased risk of dysphagia in patients with deficient nutritional status, which is a determining factor in prognosis^(24,26)^.

The institutionalized older adults in this study and those who returned to their homes were and continue to be monitored by speech-language-hearing pathologists when necessary. Their intervention helped to improve swallowing function and quality of life^(26)^ and reduce complaints^(12,23)^. Given the above, factors inherent to aging should be investigated with the help of self-assessment protocols and a trained professional to better direct care.

Participants with self-assessed risk of dysphagia who responded to the quality-of-life questionnaire (SWAL-QOL) showed a greater impact on “eating duration”. Overall, the mean scores indicate a good quality of life, even when faced with the risk of dysphagia. Modifications and adaptations during oral intake, such as not talking during meals, eating slowly, cutting food into small pieces, avoiding consistencies, and drinking water to aid swallowing solid foods^(23)^, can minimize dysphagia symptoms and be recognized as inherent to aging. Such behaviors lead to a reduced perception of impacts on quality of life. A study shows that individuals aged 18 to 89 years experience impacts on their swallowing quality of life^(22)^. Hence, it can be inferred that the symptoms described in the swallowing quality of life protocol are guiding factors in the diagnosis, treatment, and prognosis of swallowing disorders, especially in older people.

This study found that older age and functional frailty were related to self-assessed risk of dysphagia, corroborating the literature^(15,16,23,24,29,30)^. The multivariate model showed that being over 80 years old increased the chance of an older person self-assessing the risk of dysphagia by 3.78 times when compared to those aged 60 to 79 years. Also, being classified as frail increased the chance of self-assessing the risk of dysphagia by 4.55 times when compared to robust older people. A study with patients admitted to a university hospital found a high number of older people with dysphagia and a statistical association with dysphonia, gastroesophageal reflux, tracheostomy, and alternative feeding routes^(30)^. Frail adults and those with a more advanced age were more likely to be classified as frail or at risk of frailty and to have a voice handicap and restricted auditory participation^(10)^. Also, those over 60 years old, registered in the Family Health Strategy in the urban and rural areas of Cuité, Brazil, had clinical-functional vulnerability associated with advanced age, sedentary lifestyle, functional illiteracy, high stress, health problems, and medication use^(14)^.

Aging is one of the major predictors of increased frailty^(10,14,15)^ and serves as a warning for the maintenance and strengthening of public policies for the health of older adults, aiming to adopt health promotion and disease prevention measures to enhance their autonomy and independence^(14)^. Older adults’ vulnerability manifests through individual and social aspects, and its presence and intensity depend on age-related disabilities^(27)^. These findings infer the importance of speech-language-hearing therapy in public policies, focused on reducing the potential impacts caused by dysphagia and inadequate adjustments made by these patients throughout life to avoid complications such as pulmonary aspiration.

Psychosocial problems can intensify due to the public policy of compulsory isolation for leprosy treatment, coupled with the loss of functioning resulting from the disease and aging, resulting from decreased status in the community, discrimination, social exclusion, and reduced self-care activities^(1,3)^. Thus, preventing disabilities caused by the disease allows patients to maintain or improve their quality of life and physical, social, economic, and emotional condition, even after treatment. Health in older adults should be understood as their ability to meet biopsychosocial needs, regardless of age or disease^(18)^. The results do not allow us to determine the contribution of a previous history of leprosy to swallowing ability, as it did not compare them with older adults without this condition. However, the use of self-assessment instruments for these changes, such as EAT-10 and SWAL-QOL, allows for early diagnosis, indicates the need for speech-language-hearing intervention, and provides self-assessment and self-care. Studies should investigate aspects related to the risk of dysphagia, especially in older adults with chronic diseases. It is not possible to state that all older adults with leprosy have dysphagia, as iatrogenic factors, advanced age, and early diagnosis and intervention of leprosy must be considered.

Therefore, the results indicate the need for health promotion programs and prevention of the impacts of leprosy and loss of functioning. As age and the clinical-functional vulnerability index increase, the self-assessment of dysphagia risk increases, making it extremely important to include speech-language-hearing pathologists in public policies, as they are the best-equipped professionals to address dysphagia. Reducing disease severity, controlling associated morbidities, and promoting healthy aging provide enhanced care, a better quality of life, greater social inclusion, physical well-being, and improved health conditions for older people, given the increase in life expectancy.

## CONCLUSION

Being over 80 years old and considered frail increases the likelihood of older adults with a history of leprosy self-reporting their risk of dysphagia. The greatest impairment in the swallowing quality of life was related to eating duration.
