# Supplementary material for: Self-assessment of risk of dysphagia and clinical-functional vulnerability in older adults with a history of leprosy
Source: Codas. 2025 Dec 1;37(6):e20240192. doi: 10.1590/2317-1782/e20240192en (PMC12704613; doi:10.1590/2317-1782/e20240192en)
Supplement: Translation

# Translation: Autoavaliação do risco de disfagia e vulnerabilidade clínico-funcional em idosos com histórico de hanseníase

**DOI:** 10.1590/2317-1782/e20240192pt

**Authors:** Maria Clara Rocha, Aline Mansueto Mourão, Jéssica Danielle Santos de Jesus, Laélia Cristina Caseiro Vicente, Adriane Mesquita de Medeiros

**Affiliations:** 1 Programa de Pós-graduação em Ciências Fonoaudiológicas, Departamento de Fonoaudiologia, Universidade Federal de Minas Gerais – UFMG - Belo Horizonte (MG), Brasil.

**Keywords:** Hanseníase, Idoso, Fragilidade, Deglutição, Transtornos de Deglutição, Prótese Dentária, Institucionalização

## Abstract

**Objetivo:**

Relacionar a autoavaliação do risco de disfagia e a vulnerabilidade clínico-funcional, idade, sexo, dentição, escolaridade e institucionalização de idosos com histórico de hanseníase e verificar a qualidade de vida em deglutição daqueles com risco.

**Método:**

Estudo transversal com 117 idosos, sendo os critérios de inclusão idade igual ou superior a 60 anos, com histórico de hanseníase e sem histórico de transtornos mentais, comprometimento cognitivo por acidente vascular cerebral ou síndrome demencial. Foram obtidas informações como sexo, idade, escolaridade, institucionalização e dentição e utilizados protocolos: Índice de Vulnerabilidade Clínico-funcional (IVCF-20), Autoavaliação do Risco de Disfagia (EAT-10) e Autoavaliação da Qualidade de Vida em Deglutição (SWAL-QOL). Verificou-se associação entre a autoavaliação do risco de disfagia em idosos com histórico de hanseníase e as demais variáveis por meio dos testes qui-quadrado de Pearson e exato de Fisher, nível de significância de 5%.

**Resultados:**

Houve predomínio de idosas entre 80 e 99 anos, com ensino fundamental incompleto, não institucionalizados e em uso de prótese dentária. De acordo com a autoavaliação, o risco de disfagia foi encontrado em 22 idosos (18,8%) e o domínio da qualidade de vida mais comprometido foi “duração da alimentação”. Houve associação estatística do risco de disfagia com a idade e a vulnerabilidade clínico funcional.

**Conclusão:**

Ter idade acima de 80 anos e ser considerado frágil aumenta a chance do idoso com histórico de hanseníase ter autoavaliação do risco de disfagia. O maior prejuízo na qualidade de vida em deglutição foi relacionado ao domínio “duração da alimentação”.

## INTRODUÇÃO

A hanseníase é considerada uma doença infectocontagiosa crônica, dermatoneurológica, de notificação compulsória e investigação obrigatória, que afeta os nervos superficiais da pele e troncos nervosos periféricos^(1)^, incluindo os nervos cranianos^(2)^. Apresenta-se como agente etiológico o *Mycobacterium leprae* e sua transmissão ocorre principalmente pelas vias aéreas superiores, sendo observadas alterações sensitivas, motoras e autonômicas^(1)^. Possui alto poder incapacitante e acomete indivíduos de ambos os sexos e de todas as faixas etárias. O tratamento é gratuito, oferecido pelo Sistema Único de Saúde (SUS) e realizado por meio de uma combinação medicamentosa, chamada poliquimioterapia – PQT^(1)^.

O diagnóstico precoce e tratamento adequado são as principais formas de prevenir as deficiências e incapacidades físicas causadas pela doença. Entretanto, quando estas já estão instaladas, o cuidado deve ser direcionado para a manutenção ou melhora das condições físicas, socioeconômicas e emocionais desses indivíduos^(1,3)^. A Estratégia Global para Hanseníase (2016-2020), criada pela Organização Mundial de Saúde (OMS), tem como objetivo reduzir a carga global e local da hanseníase e promover a eliminação do estigma, discriminação e exclusão social existentes^(3)^.

Embora a hanseníase tenha apresentado redução no número de infectados ao longo da última década^(3)^, permanece sendo um importante problema de saúde^(4)^. Em 2020, foram registrados 127.396 mil casos no mundo, sendo o Brasil o segundo colocado no ranking mundial, perdendo apenas para a Índia^(4)^. Dados preliminares de 2022 demonstram que o Brasil diagnosticou 14.962 casos novos de hanseníase. No período de 2017 e 2021, foram diagnosticados no Brasil 119.698 casos novos de hanseníase. Desse total, 66.613 casos novos ocorreram no sexo masculino, o que corresponde a 55,7% do total. Essa predominância foi observada na maioria das faixas etárias e anos da avaliação, com maior frequência nos indivíduos entre 50 a 59 anos e com ensino fundamental incompleto (40,9%)^(4,5,6)^. A prevalência da doença está diretamente interligada com o envelhecimento ^(6)^, incapacidades físicas^(7)^, questões sociodemográficas, econômicas, sanitárias e ambientais ^(3)^ *.* Por isso, a importância da identificação de grupos vulneráveis e manejo intersetorial que garanta equidade no acesso às políticas públicas de inclusão social, serviço e educação em saúde e renda, fortalecendo as ações de controle da hanseníase^(1,3)^.

As estruturas do sistema estomatognático e trato vocal podem ser acometidas pela hanseníase, comprometendo as funções de comunicação e deglutição^(8-12)^. Estudos apontam a participação dos nervos cranianos responsáveis pelo processo de deglutição como o trigêmeo, facial, vago, acessórios espinhais e hipoglosso, sendo os mais acometidos em pessoas com hanseníase o trigêmeo e facial^(2,8)^. Além disso, a doença pode afetar a mucosa da laringe e desenvolver sintomas como tosse seca, sensação de corpo estranho na garganta, odinofagia, estridor, disfagia, disfonia, afonia e dispneia^(11-13)^. Embora, os sintomas surjam em fases mais avançadas da doença^(11,13)^, a maioria desses indivíduos apresentam deglutição eficiente^(12)^, sendo referido que os fatores iatrogênicos, idade avançada e intervenção precoce no tratamento, podem influenciar na presença e grau da disfagia^(12,13)^.

O envelhecimento e a disfagia são fatores de risco potenciais para o desenvolvimento e progressão da fragilidade^(10,14-16)^. Desse modo, ao identificar a presença de sinais e sintomas de disfagia em idosos com histórico de hanseníase, com auxílio de protocolos direcionados ^(9)^, o profissional pode contribuir para a qualidade de vida desta população, promovendo ações preventivas e de reabilitação, visando a redução de complicações como broncoaspiração e pneumonias aspirativas, desnutrição e desidratação, que levam a debilitação da saúde e prejuízos funcionais^(8-10,12)^.

O estudo tem como hipótese que, há relação entre a autoavaliação do risco de disfagia em idosos com histórico de hanseníase e à vulnerabilidade clínico funcional, idade, sexo, condição dentária, escolaridade e institucionalização. Por essa razão, faz-se necessário conhecer quem são os idosos com risco de disfagia e o impacto da deglutição na sua qualidade de vida, a fim de favorecer a ampliação do cuidado da pessoa idosa, diante das perdas funcionais decorrentes da hanseníase e do envelhecimento.

Diante disso, o objetivo desse estudo, foi analisar a relação entre a autoavaliação do risco de disfagia e a vulnerabilidade clínico-funcional, idade, sexo, dentição, escolaridade e institucionalização de idosos com histórico de hanseníase e verificar a qualidade de vida em deglutição para aqueles com risco.

## MÉTODO

Trata-se de uma pesquisa observacional analítica transversal aprovada pelos comitês de ética em pesquisa da rede Fundação Hospitalar do Estado de Minas Gerais (FHEMIG) e da UFMG sob o parecer 2.373.001. Todos os participantes deram seu consentimento por meio do Termo de Consentimento Livre e Esclarecido (TCLE).

O estudo foi realizado com 117 idosos, sendo os critérios de inclusão idade igual ou superior a 60 anos, com histórico de hanseníase e sem histórico de transtornos mentais, comprometimento cognitivo por acidente vascular cerebral (AVC) ou síndrome demencial. Os critérios de exclusão foram idosos com suspeita de alteração cognitiva de acordo com o Mini Exame do Estado Mental (MEEM), segundo o nível de escolaridade^(17)^ .

A coleta de dados foi realizada em uma instituição localizada na região metropolitana de Minas Gerais. A inauguração da instituição foi em 1931 quando a internação compulsória e o isolamento do doente eram as medidas adotadas para tratamento e profilaxia da hanseníase, deixando de ocorrer dessa forma no final da década de 80. A maioria dos idosos participantes desse estudo vivem em domicílios próprios atualmente e os idosos mais fragilizados e os que perderam os vínculos familiares e sociais vivem na instituição de longa permanência para idosos (ILPI) da instituição. São ofertados atendimentos especializados nos níveis ambulatorial, domiciliar e ILPI, com a missão de cuidar principalmente dos idosos com histórico de hanseníase provenientes do período de internação compulsória. A equipe é composta por fonoaudiólogo, fisioterapeuta, terapeuta ocupacional, enfermagem, serviço social, psicologia e nutrição.

A coleta dos dados primários da pesquisa se deu em duas etapas, sendo ambas no mesmo período de coleta. A primeira por meio de extração dos dados atuais nos prontuários da instituição, incluindo informações pessoais como idade, sexo, edentulismo, adaptação de próteses dentárias e institucionalização. A segunda etapa, por meio da aplicação de protocolos selecionados. Todos os participantes da pesquisa foram avaliados pela fonoaudióloga da equipe de reabilitação da instituição.

Utilizou-se como instrumento o Índice de Vulnerabilidade Clínico-Funcional (IVCF-20), questionário validado no Brasil, que considera aspectos multidimensionais da condição de saúde dos idosos. O protocolo é constituído por 20 questões distribuídas em oito seções: idade, autopercepção da saúde, incapacidades funcionais, cognição, humor, mobilidade, comunicação e comorbidades múltiplas. Cada seção tem pontuação específica que totalizam um valor máximo de 40 pontos. Quanto mais alto o valor obtido, maior é a vulnerabilidade clínico-funcional do idoso. A classificação do idoso pelo IVCF-20 é apresentada em três categorias: idoso robusto, aquele que obtiver pontuação total entre 0-6; idoso em risco de fragilização, pontuação entre 7-14; idoso frágil, pontuação igual ou superior a 15^(18)^. A aplicação do IVCF-20 faz parte da rotina do serviço de reabilitação da instituição, sendo este aplicado por uma equipe composta por Fonoaudiólogo, Fisioterapeutas, Terapeutas Ocupacionais, Psicólogos, Assistentes Sociais, Nutricionistas e Enfermeiros. Os avaliadores passaram por capacitação prévia, em 2016, com a equipe que participou da criação do IVCF-20. Os dados desse protocolo que foram incluídos no estudo se referiam às avaliações realizadas no mesmo período que se realizou a coleta dos dados primários dessa pesquisa.

Em seguida, foi realizada a aplicação do protocolo de autoavaliação do risco de disfagia e qualidade de vida relacionada à deglutição. A versão em português do *Eating Assessment Tool* (EAT-10)^(19,20)^ visa identificar a autoavaliação do risco para disfagia e é composto por dez questões simples, três do domínio funcional, três do domínio emocional e quatro do domínio físico, sendo a pontuação total máxima de 40 pontos. O valor igual ou superior a 3 pontos, indica o risco de disfagia, sendo o cálculo simples e realizado por meio da soma dos resultados de cada item.

Para identificar o impacto da autoavaliação do risco de disfagia na qualidade de vida, aplicou-se o instrumento de autoavaliação *Quality of Life in Swallowing Disorders* – SWAL-QOL^(21)^ na versão validada para o português^(22)^ nos idosos considerados com risco de disfagia segundo o EAT-10. Este questionário é composto por 44 questões que avaliam onze domínios, sendo eles: deglutição como um fardo, desejo de se alimentar, duração da alimentação, frequência de sintomas, seleção de alimentos, comunicação, medo de se alimentar, saúde mental, social, sono e fadiga. As respostas relacionadas à frequência e grau de veracidade são marcadas por meio da Escala de Likert e variam de 1 a 5, sendo atribuídos os seguintes pontos: 1=0, 2=25, 3=50, 4=75 ou 5=100. A pontuação varia de 0 a 100, sendo que quanto mais baixa, pior a qualidade de vida relacionada à deglutição. Os valores de cada resposta dentro de cada domínio são somados, e o resultado é dividido pelo número de questões do domínio analisado. A pontuação final é resultante da soma dos valores obtidos na pontuação em cada domínio dividido pelo número total de domínios.

Quanto à análise de dados, esta ocorreu de forma descritiva por meio da distribuição de frequência absoluta e relativa. Para analisar a associação da autoavaliação do risco de disfagia com as variáveis explicativas, foi usado o teste qui quadrado de Pearson e exato de Fisher. As variáveis que apresentaram significância estatística nos testes de associação (idade e vulnerabilidade clínico-funcional) foram incluídas na análise univariada de regressão logística para estimar a razão de chance (*odds ratio*). Para todas as análises foi considerado o nível de significância de 5%. Os dados foram tratados e analisados no programa STATA versão 13.

## RESULTADOS

O estudo foi realizado com 117 idosos homens e mulheres, sendo observado predomínio de idosos do sexo feminino (54,7%), com idades entre 80 e 99 anos (52,99%) e ensino fundamental incompleto (75,2%). Dos indivíduos participantes, 18 (15,4%) são institucionalizados, 90 (77,0%) não possuem os dentes naturais e 44 (37,6%) fazem uso de próteses dentárias, sejam elas parciais ou totais. Quanto à vulnerabilidade funcional, 37,6% dos idosos foram considerados robustos, 35,0% em risco de fragilização e 27,5% frágeis (Tabela 1).

**Tabela 1:** Descrição das características sociodemográficas, condições dentárias e índice de vulnerabilidade social dos idosos com histórico de hanseníase (n=117)

Variáveis	Idosos com risco de disfagia	Idosos sem risco de disfagia	Total	Valor - *p*
	n (%)	n (%)		
**Idade**				
64 a 79 anos	5 (22,7)	50 (52,6)	55 (47,0)	0,011*^a^
80 a 99 anos	17 (77,3)	45 (47,4)	62 (53,0)	
**Sexo**				
Feminino	10 (45,5)	54 (56,8)	64 (54,7)	0,33*
Masculino	12 (54,5)	41 (43,2)	53 (45,3)	
**Institucionalização**				
Sim	5 (22,7)	13 (13,7)	18 (15,4)	0,29*
Não	17 (77,3)	82 (86,3)	99 (84,6)	
**Escolaridade**				
Nenhuma Escolaridade	7 (31,8)	17 (17,9)	24 (20,5)	0,55**
Ensino Fundamental incompleto	15 (68,2)	73 (76,8)	88 (75,2)	
Ensino Fundamental completo	0 (0,0)	1 (1,1)	1 (0,9)	
Ensino Médio incompleto e completo	0 (0,0)	4 (4,2)	4 (3,4)	
**Dentes Naturais**				
Sim	7 (31,8)	20 (21,1)	27(23,1)	0,28*
Não	15 (68,2)	75 (78,9)	90 (76,9)	
**Prótese dentária bem adaptada**				
Sim	5 (22,7)	39 (41,1)	44 (37,6)	0,13*
Não	12 (54,5)	31 (32,6)	43 (36,8)	
Não usa prótese dentária	5 (22,7)	25 (26,3)	30 (25,6)	
**Índice de Vulnerabilidade Clínico-Funcional (IVCF-20)**				
Robusto	4 (18,2)	40 (42,1)	44 (37,6)	0,05*^a^
Em risco de fragilização	8 (36,4)	33 (34,7)	41 (35,0)	
Frágil	10 (45,4)	22 (23,2)	32 (27,4)	

* Teste qui-quadrado de Pearson;

** Teste exato de Fisher

a valor-p≤0,05

Verificou-se que somente as variáveis idade e o IVCF-20 apresentaram associação nos idosos com risco de disfagia (Tabela 1).

Segundo o EAT-10 foi observado a autoavaliação do risco de disfagia em 22 idosos (18,8%), sendo os sintomas mais relatados “preciso fazer força para engolir remédios”, “fico com comida presa entalada na garganta” e “eu tusso quando como” (Tabela 2).

**Tabela 2:** Descrição das respostas do EAT-10 (n=117)

Variáveis	Total
n (%)	
**Meu problema para engolir me faz perder peso**	
Não é um problema	115 (98,30%)
Problema pequeno	0 (0,00%)
Problema médio	2 (1,70%)
Problema grande	0 (0,00%)
Problema muito grande	0 (0,00%)
**Meu problema para engolir não me deixa comer fora de casa**	
Não é um problema	110 (94,00%)
Problema pequeno	0 (0,00%)
Problema médio	3 (2,60%)
Problema grande	2 (1,70%)
Problema muito grande	2 (1,70%)
**Preciso fazer força para engolir líquidos**	
Não é um problema	109 (93,15%)
Problema pequeno	1 (0,85%)
Problema médio	5 (4,30%)
Problema grande	1 (0,85%)
Problema muito grande	1 (0,85%)
**Preciso fazer força para engolir comidas (sólidos)**	
Não é um problema	105 (89,75%)
Problema pequeno	1 (0,85%)
Problema médio	6 (5,15%)
Problema grande	4 (3,40%)
Problema muito grande	1 (0,85%)
**Preciso fazer força para engolir remédios**	
Não é um problema	95 (81,20%)
Problema pequeno	0 (0,00%)
Problema médio	18 (15,40%)
Problema grande	3 (2,55%)
Problema muito grande	1 (0,85%)
**Dói para engolir**	
Não é um problema	115 (98,30%)
Problema pequeno	0 (0,00%)
Problema médio	2 (1,70%)
Problema grande	0 (0,00%)
Problema muito grande	0 (0,00%)
**Meu problema para engolir me tira o prazer de comer**	
Não é um problema	113 (96,60%)
Problema pequeno	0 (0,00%)
Problema médio	3 (2,55%)
Problema grande	1 (0,85%)
Problema muito grande	0 (0,00%)
**Fico com comida presa entalada na garganta**	
Não é um problema	99 (84,61%)
Problema pequeno	4 (3,41%)
Problema médio	8 (6,83%)
Problema grande	4 (3,41%)
Problema muito grande	2 (1,70%)
**Eu tusso quando como**	
Não é um problema	89 (76,05%)
Problema pequeno	2 (1,70%)
Problema médio	14 (12,00%)
Problema grande	9 (7,70%)
Problema muito grande	3 (2,55%)
**Engolir me deixa estressado**	
Não é um problema	113 (96,60%)
Problema pequeno	0 (0,00%)
Problema médio	4 (3,40%)
Problema grande	0 (0,00%)
Problema muito grande	0 (0,00%)
**Risco de disfagia - EAT**	
Sim (ponto de corte: >2 pontos)	22 (18,80%)
Não (ponto de corte até 2 pontos)	95 (81,20%)

**Legenda:** EAT-10 = *Eating Assessment Tool*

Todos os participantes considerados com risco de disfagia, responderam ao questionário de qualidade de vida (SWAL-QOL). Pode-se identificar, que as maiores médias dos escores são os domínios Comunicação (88,0%) e Social (86,8%), indicando não ser um problema para a qualidade de vida em deglutição dos idosos com autoavaliação para o risco de disfagia. Já o maior prejuízo na qualidade de vida em deglutição foi referente ao tempo de alimentação (19,3%). A menor média nesse escore mostrou que os idosos demoram mais tempo para comer do que outras pessoas e percebem que demoram muito para comer uma refeição (Tabela 3).

**Tabela 3:** Descrição da mediana, média e desvio-padrão dos escores referente ao Swal-QOL dos idosos com autoavaliação do risco de disfagia

Domínios	Idosos com risco de disfagia	Média (desvio-padrão)
	Mediana	
Deglutição como fardo	62,5	61,9 (28,2)
Desejo de se alimentar	66,7	64,4 (29,5)
Duração da alimentação	0	19,3 (32,4)
Frequência de sintomas	79,5	74,4 (10,9)
Seleção dos alimentos	81,3	79,5 (23,0)
Comunicação	100,0	88,0 (21,2)
Medo de se alimentar	62,5	65,0 (26,8)
Saúde mental	80,0	73,8 (21,6)
Social	92,5	86,8 (16,2)
Sono	56,3	63,0 (26,5)
Fadiga	75,0	73,8 (17,1)
Escore Total do SWAL-QOL	71,9	67,9 (10,6)

**Legenda:** Swal-QOL = *Quality of Life in Swallowing Disorders*

Na análise univariada de regressão logística, foi confirmada a associação estatística entre a autoavaliação do risco de disfagia com a idade e vulnerabilidade clínico-funcional (Tabela 4).

**Tabela 4:** Modelo univariado de regressão logística da associação da autoavaliação do risco de disfagia com as variáveis idade e IVCF-20

	OR (IC95%)
**Idade**	
64 a 79 anos	1
80 a 99 anos	3,78 (1,29-11,07)*
**IVCF-20**	
Robusto	1
Em risco de fragilização	2,42 (0,67-8,77)
Frágil	4,55 (1,28-16,20)*

* valor-p≤0,05

**Legenda:** IVCF-20: Índice de Vulnerabilidade Clínico-Funcional; OR = Odds Ratio; IC95% = Intervalo de confiança de 95%

O resultado mostra que o idoso frágil tem 4,55 mais chance de ter autoavaliação do risco de disfagia quando comparado àqueles com índice robusto de funcionalidade clínico-funcional. E os idosos mais velhos, possuem 3,78 mais chance de ter autoavaliação do risco para a disfagia (Tabela 4). Não foi realizado o modelo multivariado com as duas variáveis que apresentaram significância estatística, pois a análise entre idade e o IVCF-20 mostrou relação entre a vulnerabilidade funcional do idoso e a idade. Idosos com idade entre 80 e 99 anos tem mais chance de estar em risco de fragilização (OR=2,73/ IC95%=1,13-6,58) e em situação de fragilidade (OR=4,94/IC95%=1,83-13,31) quando comparados aos com idade de 64 a 79 anos.

## DISCUSSÃO

O presente estudo mostrou que, a maior parte dos idosos com histórico de hanseníase não apresentou autoavaliação para o risco de disfagia. Contudo, a autoavaliação do risco de disfagia foi associada com a idade e ao maior índice de vulnerabilidade clínico-funcional medida pelo IVCF-20, sendo o domínio da qualidade de vida em deglutição mais comprometido foi “duração da alimentação”.

Dados epidemiológicos atuais no Brasil indicam a maior prevalência de hanseníase em adultos^(4)^, na faixa etária próxima do idoso^(6)^ o que contribui para o aumento da suscetibilidade à alterações orais e laringofaríngeas^(12,13)^, bem como a vulnerabilidade clínico funcional^(10)^ nessa população. Apesar dos idosos terem realizado diagnóstico e tratamento da hanseníase no passado, previamente à terceira idade, sabe-se da convivência com as sequelas crônicas decorrentes da doença^(1,3)^ e, consequente perda da funcionalidade devido ao processo de envelhecimento^(7,15)^.

Houve predomínio de idosos do sexo feminino, com idades entre 80 e 99 anos e nível de escolaridade reduzido, corroborando a literatura^(7,23,24)^. Não houve associação estatística da autoavaliação do risco de disfagia com o sexo e a escolaridade no presente estudo, como observado em outras pesquisas, em que não encontraram essa relação com o sexo em idosos saudáveis^(25)^ e com a escolaridade em idosas institucionalizadas^(26)^. Em contrapartida, observa-se na literatura, a ocorrência de disfagia em mulheres com baixa escolaridade^(15,24)^. Tal achado pode ser explicado devido a maior procura da população feminina aos serviços de saúde e aos aspectos culturais, comportamentais, de educação e hábitos de vida envolvidos^(27)^.

O protocolo EAT-10, mostrou que 18,8% dos idosos com histórico de hanseníase, apresentaram autoavaliação do risco de disfagia, valor abaixo de outro estudo com população similar, mas com avaliação clínica da deglutição^(12)^ .

Achados relacionados à disfagia em idosos sem o diagnóstico de hanseníase, verificou risco de disfagia em indivíduos independentes (25,1%), dependentes (53,8%)^(28)^, saudáveis (38,5%)^(25)^, hospitalizados (31,1%)^(29)^ e frágeis (58%)^(15)^. O que explica que, apesar do elevado número de pacientes com histórico de hanseníase e deglutição eficiente, muitos idosos, independente de suas condições clínicas, apresentam disfagia leve a moderada ^(12)^, podendo estar associada ao envelhecimento e ocorrência de processos patológicos^(15,30)^. Diferentes protocolos e classificações para avaliar a disfagia podem justificar as diferenças encontradas entre os estudos. Todavia, o diagnóstico e tratamento precoce da doença, reduz os agravos e a perda da funcionalidade, podendo explicar o resultado.

Dos participantes, a maioria não possui os dentes naturais, sendo que destes, poucos fazem uso de próteses dentárias. Apesar do estudo não apresentar associação estatística entre a autoavaliação do risco de disfagia e as condições dentárias, estudos apontam relação entre a disfagia e o número de dentes^(15,26)^. Idosos coreanos apresentaram risco de disfagia de 61,6%, sendo um dos fatores associados, a menor capacidade de mastigação^(24)^. Já em uma pesquisa realizada com idosos institucionalizados, verificou-se tempo de mastigação aumentado^(23)^. Ou seja, a perda de dentição natural, o uso de próteses mal adaptadas^(15,23,24,26)^ e as lesões dos nervos cranianos responsáveis por tal função^(2,8)^ podem interferir no preparo oral do bolo alimentar e no processo de deglutição, trazendo impactos para a digestão, comunicação, interação social e qualidade de vida.

No presente estudo, todos os participantes da pesquisa, passaram pela política pública de isolamento compulsório para tratamento da hanseníase e, após o término da obrigatoriedade do isolamento, muitos retornaram para suas próprias residências, retomando assim, as atividades habituais do cotidiano. Não houve significância estatística entre a autoavaliação do risco de disfagia em idosos institucionalizados. No entanto, em uma pesquisa realizada com 23 idosos brasileiros institucionalizados, observou-se alterações de deglutição em 82,6% dos participantes, associadas ao envelhecimento^(23)^. Idosas institucionalizadas de outra pesquisa, apresentaram risco de disfagia de 63,3%, relacionado a fatores como uso de medicamentos, número de dentes presentes e dinâmica alimentar^(26)^. Mesmo diante a ausência de resultados nesta pesquisa em relação ao estado nutricional, a literatura aponta aumento do risco de disfagia em pacientes em estado nutricional deficiente, sendo determinante no prognóstico^(24,26)^.

Os idosos institucionalizados deste estudo, bem como os que retornaram para seus domicílios, foram e são acompanhados pelo profissional fonoaudiólogo, quando necessário. A intervenção fonoaudiológica contribuiu para melhora da função de deglutição, para a qualidade de vida^(26)^ e redução das queixas^(12,23)^. Diante o exposto, os fatores inerentes ao envelhecimento devem ser investigados com auxílio dos protocolos de autoavaliação e do profissional capacitado para melhor direcionamento do cuidado.

Os participantes com autoavaliação do risco de disfagia que responderam ao questionário de qualidade de vida (SWAL-QOL), apresentaram maior impacto no domínio “duração da alimentação”. De modo geral, pode-se observar por meio das médias obtidas por escores, valores correspondentes a uma boa qualidade de vida, mesmo diante o risco de disfagia. Modificações e adaptações durante a ingesta oral como: não falar durante a alimentação, comer devagar, cortar a comida em pedaços pequenos, evitar consistências e beber água para auxiliar a deglutição de alimentos sólidos^(23)^, podem minimizar os sintomas da disfagia e serem reconhecidas como inerentes ao envelhecimento. Tais comportamentos levam a redução da percepção dos impactos na qualidade de vida. Estudo mostra que indivíduos com idades entre 18 e 89 anos, apresentam impactos na qualidade de vida em deglutição^(22)^. Sendo assim, podemos inferir que, os sintomas descritos no protocolo de qualidade de vida em deglutição, são norteadores no processo de diagnóstico, terapêutica e prognóstico dos distúrbios de deglutição, principalmente na pessoa idosa.

A relação entre a idade mais avançada e fragilidade funcional com a autoavaliação do risco de disfagia foi observada nesse estudo, corroborando a literatura^(15,16,23,24,29,30)^. O modelo multivariado mostrou que ter idade acima de 80 anos aumentou em 3,78 vezes a chance de o idoso ter autoavaliação do risco de disfagia quando comparado aos com idade entre 60 e 79 anos. E ser classificado como frágil aumentou 4,55 vezes a chance de autoavaliação do risco de disfagia quando comparado aos idosos robustos. Em pesquisa realizada com pacientes internados em um hospital universitário, verificou-se número elevado de idosos com disfagia e associação estatística com a presença de disfonia, refluxo gastroesofágico, uso de traqueostomia e via alternativa de alimentação^(30)^. Idosos mais velhos e frágeis, tiveram mais chance de serem classificados em risco de fragilização ou frágil e apresentarem desvantagem vocal e restrição à participação auditiva^(10)^. Já outros com idade superior a 60 anos, cadastrados na Estratégia Saúde da Família da zona urbana e/ou rural do município de Cuité tiveram a vulnerabilidade clínico-funcional associada a idade avançada, sedentarismo, analfabetismo funcional, estresse elevado, problemas de saúde e uso de medicamentos^(14)^.

O envelhecimento é um dos grandes preditores para o aumento da fragilidade^(10,14,15)^, sendo um alerta para a manutenção e fortalecimento das políticas públicas voltadas à saúde do idoso, para que sejam adotadas medidas de promoção da saúde e prevenção de agravos no sentido de potencializar a autonomia e independência desses indivíduos^(14)^. A vulnerabilidade da pessoa idosa se apresenta por meio de aspectos individuais e sociais e sua presença e intensidade vão depender das incapacidades advindas da idade^(27)^. Tais achados, inferem a importância da atuação fonoaudiológica nas políticas públicas, com enfoque na redução dos possíveis impactos causados pela disfagia e ajustes inadequados realizados por esses indivíduos ao longo da vida, a fim de evitar complicações como a broncoaspiração.

Devido a política pública de isolamento compulsório para tratamento da hanseníase, juntamente com a perda da funcionalidade decorrente da doença e do processo de envelhecimento, problemas psicossociais podem se intensificar, fruto da diminuição do status na comunidade, bem como discriminação, exclusão social e redução das atividades relacionadas ao autocuidado^(1,3)^. Por isso, a prevenção das incapacidades causadas pela doença, proporcionam ao paciente mesmo após o tratamento, a manutenção ou melhora de sua condição física, social, econômica, emocional e da qualidade de vida. A saúde no idoso deve ser compreendida como a capacidade individual de satisfação das necessidades biopsicossociais, independentemente da idade ou da presença de doenças^(18)^. Os resultados não permitem afirmar qual a contribuição da história pregressa de hanseníase sobre a condição de deglutição pela ausência de comparação com os idosos sem essa condição. Entretanto, a utilização de instrumentos de autoavaliação dessas alterações, como o protocolo de risco de disfagia (EAT-10) e de qualidade de vida em deglutição (SWAL-QOL) proporcionam o diagnóstico precoce e necessidade de intervenção fonoaudiológica, bem como a autoavaliação e autocuidado desses indivíduos. Estudos devem procurar verificar os aspectos relacionados ao risco de disfagia, principalmente em idosos com doenças crônicas. Não é possível afirmar que todo idoso com hanseníase possui disfagia, pois deve-se considerar os fatores iatrogênicos, idade avançada e diagnóstico e intervenção precoce da hanseníase.

Sendo assim, os resultados encontrados, indicam a necessidade de programas de promoção da saúde e prevenção dos impactos causados pela hanseníase e perda da funcionalidade. Conforme a idade e o índice de vulnerabilidade clínico-funcional aumenta, a autoavaliação do risco de disfagia se eleva, sendo de extrema importância a implementação do fonoaudiólogo nas políticas públicas, visto que é o profissional capacitado para a intervenção das disfagias. A redução dos agravos da doença, controle das morbidades associadas e o favorecimento do envelhecimento saudável, proporcionam o cuidado ampliado, melhor qualidade de vida, maior inserção social, bem-estar físico e melhores condições de saúde da população idosa, diante do aumento da expectativa de vida.

## CONCLUSÃO

Ter idade acima de 80 anos e ser considerado frágil aumenta a chance do idoso com histórico de hanseníase ter autoavaliação do risco de disfagia. O maior prejuízo na qualidade de vida em deglutição foi relacionado ao domínio “duração da alimentação.
